# Necessity as a Function of Skewness

**DOI:** 10.3389/fpsyg.2017.02192

**Published:** 2017-12-19

**Authors:** Kimmo Sorjonen, Jenny Wikström Alex, Bo Melin

**Affiliations:** Division of Psychology, Department of Clinical Neuroscience, Karolinska Institute, Solna, Sweden

**Keywords:** creativity, intelligence, necessity, simulation, skewness

## Abstract

With necessary condition analysis (NCA), a necessity effect is estimated by calculating the amount of empty space in the upper left corner in a plot with a predictor X and an outcome Y. In the present simulation study, calculated necessity effects were found to have a negative association with the skewness of the predictor and a positive association with the skewness of the outcome. Also the standard error of the necessity effect was found to be influenced by the skewness of the predictor and the skewness of the outcome, as well as by sample size, and a way to calculate a confidence interval for the necessity effect is presented. At least some of the findings obtained with NCA are well within the range of what can be expected from the skewness of the predictor and the outcome alone.

## Introduction

**FIGURE 1:**
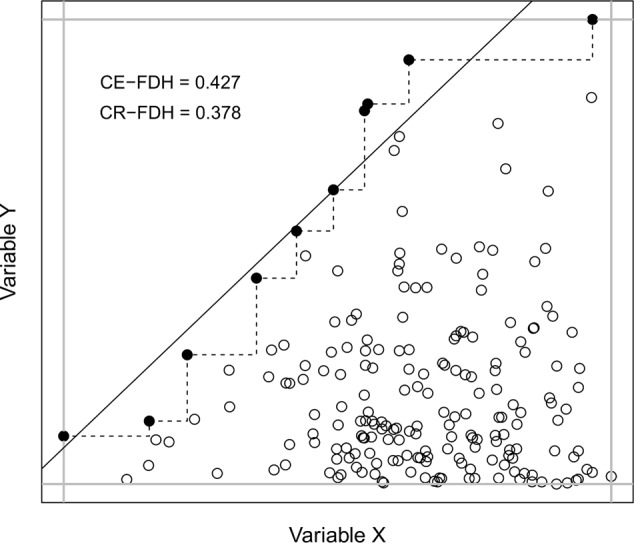
An illustration of NCA, where the size of the empty/semi-empty upper-left corner is calculated using either a step function (CE-FDH, dotted line) or a linear regression function (CR-FDH, solid line). In this example, *N* = 200, skewness of *X* = –0.60, and skewness of *Y* = 1.52.

Dul (2016a) has developed necessary condition analysis (NCA) as a tool to investigate whether a factor X can be considered a necessary condition for another factor Y. In this analysis one simply calculates the amount of empty space in the upper-left corner when plotting X and Y against each other (**Figure 1**). A large empty space in the upper-left corner is taken to indicate that it is impossible to achieve a high value on Y with a low value on X, i.e., that a certain minimum level of X is necessary for a high level of Y. Either a step function (CE-FDH) or a linear regression function (CR-FDH) is applied to ceiling points with a higher y-value than all points with a lower x-value (black dots in **Figure 1**). According to Dul (2016a), the degree of necessity is calculated by dividing the area of the empty/semi-empty upper-left corner with the area given by (X_max_ – X_min_) × (Y_max_ – Y_min_) (inner square with gray border in **Figure 1**). In **Figure 1**, the calculated necessity effects tells us that 42.7% of this square is above/to the left of the dotted line given by a step function (=CE-FDH) and 37.8% is above/to the left of the solid regression line (=CR-FDH). According to Dul (2016a), CE-FDH and CR-FDH values below 0.1 could be characterized as small, values between 0.1 and 0.3 as medium, values between 0.3 and 0.5 as large, and values above 0.5 as very large necessity effects.

Necessary condition analysis has been used by Karwowski et al. (2016, 2017) and based on these studies the authors concluded that a not too low level of intelligence is necessary for creativity and the same conclusion was drawn by Shi et al. (2017). Other researchers using NCA have concluded that a certain degree of workplace spirituality is necessary for high levels of employee commitment, job satisfaction, and work-life balance satisfaction (Garg, 2017), that contracts with at least medium levels of contractual detail as well as the highest levels of trust are necessary for buyer–supplier relationships that have high levels of innovation (Van der Valk et al., 2016), and that a certain minimum level of safety consciousness is required to achieve top productivity results among long-haul truck drivers (De Vries et al., 2017).

Reading the literature mentioned above, we started to suspect that the degree of necessity, as quantified by NCA, might be influenced by the skewness of the predictor and of the outcome, and that the precision of the necessity effect (i.e., its standard error) might also be influenced by sample size. The objective of the present simulation study was to explore these possibilities.

## Method

Data was simulated and analyzed using R 3.3 (R Core Team, 2016). R is open source software that can be used for statistical analyses. Researchers and others have created and made available s.c. packages for specific analyses that can be used together with R. Two of these packages, namely one called “NCA” (Dul, 2016b) and another one called “moments” (Komsta and Novomestky, 2015), were used in the present study. Data was simulated through the following steps (script and data available as Supplementary Material): (1) variables X and Y of a defined length were randomly drawn from beta distributions with varying degrees of skewness; (2) the degree of necessity, as quantified by NCA (both CE-FDH and CR-FDH), as well as the skewness of X and Y were calculated and saved in a data frame. These steps were repeated a 1000 times for each combination of 5 sample sizes, 7 defined population skewness of X, and 7 defined population skewness of Y, resulting in 245,000 data sets. The used sample sizes were 50, 200, 800, 3200, and 12.800. Variables X and Y were drawn from beta distributions with (a) alpha = 9 and beta = 1; (b) alpha = 9 and beta = 2; (c) alpha = 9 and beta = 3; (d) alpha = 9 and beta = 9; (e) alpha = 3 and beta = 9; (f) alpha = 2 and beta = 9; (g) alpha = 1 and beta = 9, which corresponds to skewness (a) -1.47; (b) -0.88; (c) -0.59; (d) 0; (e) 0.59; (f) 0.88; and (g) 1.47.

## Results

In order to always predict a value between 0 and 1, CE-FDH and CR-FDH were transformed to log odds (logit) and these were highly predictable [*R*^2^ = 0.70 and 0.64 for logit(CE-FDH) and logit(CR-FDH), respectively] from the skewness of X and Y and their interaction according to the following formulas:

$$\begin{array}{c} \log(CE-FDH/(1-CE-FDH))\;=\;-3.19\;-\;1.14\;\times \;\mathrm{Skew}(X)+\;1.14\;\times \;\mathrm{Skew}(Y)+\;0.10\;\times \;\mathrm{Skew}(X)\;\times \;\mathrm{Skew}(Y)\\ \log(CR-FDH/(1-CR-FDH))\;=\;-3.02\;-\;0.88\;\times \;\mathrm{Skew}(X)+\;0.95\;\times \;\mathrm{Skew}(Y)\;-0.027\;\times \;\mathrm{Skew}(X)\;\times \;\mathrm{Skew}(Y) \end{array}$$

The standard error of CE-FDH and CR-FDH was calculated for various combinations of sample size, skewness of X, and skewness of Y, and the natural logarithm (in order to never predict a negative standard error) of these were highly predictable [*R*^2^ = 0.71 and 0.87 for log(*SE*(CE-FDH)) and log(*SE*(CR-FDH)), respectively] according to the following formulas:

$$\begin{array}{c} \log(SE(CE-FDH))\;=\;-3.14-0.013\;\times \;\mathrm{sqrt}(N)\;-0.67\times \;\mathrm{Skew}(X)\;+\;0.65\;\times \;\mathrm{Skew}(Y)\\ \log(SE(CR-FDH))\;=\;-3.09-\;0.0078\;\times \;\mathrm{sqrt}(N)-0.40\;\times \;\mathrm{Skew}(X)\;+\;0.48\;\times \;\mathrm{Skew}(Y) \end{array}$$

**FIGURE 2:**
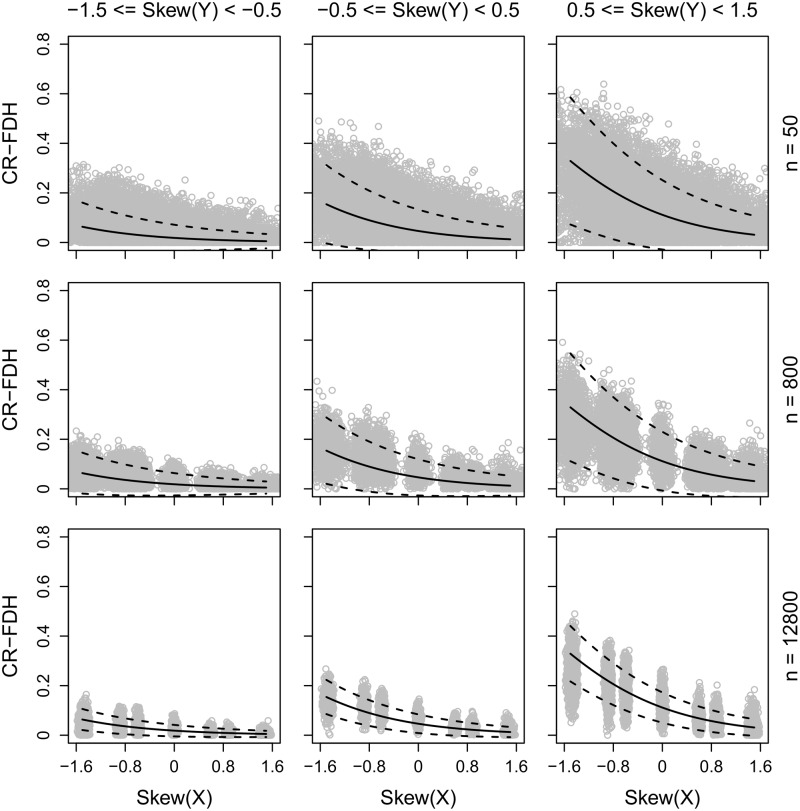
CR-FDH (with 95% CI) as a function of the skewness of X, separately for the combination of three different ranges of the skewness of Y and three different sample sizes.

The association between the skewness of X and the degree of necessity (CR-FDH) for the combinations of three different ranges of skewness of Y and three different sample sizes, is presented in **Figure 2**. The predicted CR-FDH, as given by the formula above, is included as a solid line in the plots. According to the z- and t-distributions, 95% of parameter values calculated in samples are expected to be within two standard errors from the corresponding parameter value in the population that the sample has been drawn from (Argyrous, 2000). Therefore, by adding and subtracting two predicted standard errors to/from the predicted CR-FDH, we get a predicted 95% CI and these are included as dotted lines in the plots.

A NCA of these results obtained through NCA (CR-FDH only) revealed a large effect of the skewness of X (CE-FDH = 0.349, CR-FDH = 0.341) and of the skewness of Y (CE-FDH = 0.366, CR-FDH = 0.357) but not of sample size (CE-FDH and CR-FDH = 0). A bottleneck analysis (available in NCA) showed that a large necessity effect (CR-FDH > 0.3) requires the skewness of X to be below -0.9 and the skewness of Y to be above 0.9 (**Table 1**).

**Table 1:** Bottlenecks required to achieve three levels of CR-FDH.

CR-FDH	Skew(X)	Skew(Y)
0.1	–0.098	0.186
0.3	–0.915	0.932
0.5	–1.732	1.678

## Discussion

According to the present simulation, the probability of getting a result indicating a high degree of necessity, as quantified by NCA, increases with a negatively skewed predictor X and with a positively skewed outcome Y. Both the skewness of X and the skewness of Y had large necessity effects on the calculated necessity effect. The reason for this is that according to NCA, a high degree of necessity is indicated by a large empty upper-left corner in a X–Y-plot and the probability for this is given by:

$$\begin{array}{c} P(\mathrm{Necessity}\;=\;\mathrm{high})\;=\;1\;-\;P(X\;=\;\mathrm{low})\;\times \;P(Y\;=\;\mathrm{high}) \end{array}$$

As the probability of a relatively low value is lower on negatively skewed variables, while the probability of a relatively high value is lower on positively skewed variables, the probability of finding a large empty upper-left corner is highest for the combination of a negatively skewed X and a positively skewed Y.

In order not to draw hasty conclusions about necessity, researchers using NCA should be aware of this influence of skewness. It is recommended to estimate the degree of necessity that can be expected from the amount of skewness alone, for example by using the formulas presented in this paper (a function is available in the Supplementary Script), and evaluate if the observed necessity effect is substantially above this expected value, preferably outside the calculated confidence interval. For example, Karwowski et al. (2017) calculated CE-FDH = 0.25 and CR-FDH = 0.21 for the association between intelligence, as measured by Raven Progressive Matrices (RPM), and creative achievement, measured with Creative Achievement Questionnaire (CAQ), in a sample of 1594 persons. Karwowski et al. (2017) also calculated CE-FDH = 0.35 and CR-FDH = 0.30 for the association between WISC-scores and CAQ in a sample of 255 participants. These values can be compared with expected values in **Table 2**, calculated with the formulas presented in this paper and using skewness of *Y* = 2.2 as this was the skewness of CAQ mentioned in Karwowski and colleagues paper. It is apparent that unless RPM and WISC were quite severely positively skewed, and they did not seem to be according to descriptive statistics and figures, the necessity effects found by Karwowski and colleagues are well within the range of what can be expected from skewness alone. Thus, based on the findings in Karwowski et al. (2017) and the present simulation, it would be problematic to assume that intelligence is any more necessary for creative achievement than a random variable with the same skewness as intelligence is for another random variable with the same skewness as creative achievement.

**Table 2:** Expected CE-FDH and CR-FDH for five different skewness of X when skewness of *Y* = 2.2.

	Expected		95% CI |*N* = 1594		95% CI |*N* = 255	
Skew(X)	CE-FDH	CR-FDH	CE-FDH	CR-FDH	CE-FDH	CR-FDH
–1.0	0.56	0.50	0.13; 0.98	0.21; 0.79	–0.02; 1.13	0.15; 0.85
–0.5	0.44	0.39	0.14; 0.75	0.15; 0.62	0.03; 0.86	0.10; 0.67
0.0	0.33	0.28	0.12; 0.55	0.09; 0.48	0.04; 0.63	0.05; 0.52
0.5	0.24	0.20	0.09; 0.40	0.04; 0.36	0.03; 0.45	0.01; 0.39
1.0	0.17	0.13	0.06; 0.28	0.00; 0.26	0.02; 0.32	–0.02; 0.29

*The 95% CI for two different sample sizes are also given.*

The present simulation shows that with skewed variables NCA might give an unwarranted indication that a certain variable X is necessary for another variable Y, which could be seen as a threat against the usefulness and rationale of the method. We suggest that calculations of expected necessity effects, with confidence intervals, as demonstrated in the present paper, will provide at least some protection against unjustified conclusions.

## Author Contributions

All authors conceived of the presented idea. KS carried out the simulations and analyses and wrote an initial draft. All authors discussed the results and contributed to the final manuscript.

## Conflict of Interest Statement

The authors declare that the research was conducted in the absence of any commercial or financial relationships that could be construed as a potential conflict of interest.
